# Thermally nucleated magnetic reversal in CoFeB/MgO nanodots

**DOI:** 10.1038/s41598-017-16911-3

**Published:** 2017-12-01

**Authors:** Andrea Meo, Phanwadee Chureemart, Shuxia Wang, Roman Chepulskyy, Dmytro Apalkov, Roy W. Chantrell, Richard F. L. Evans

**Affiliations:** 1 Department of Physics, University of York, Heslington, York, YO10 5DD United Kingdom; 2 Computational and experimental magnetism group, Department of Physics, Mahasarakham University, Mahasarakham, Thailand; 3 Samsung Electronics, Semiconductor R&D Center (Grandis), San Jose, CA 95134 USA

## Abstract

Power consumption is the main limitation in the development of new high performance random access memory for portable electronic devices. Magnetic RAM (MRAM) with CoFeB/MgO based magnetic tunnel junctions (MTJs) is a promising candidate for reducing the power consumption given its non-volatile nature while achieving high performance. The dynamic properties and switching mechanisms of MTJs are critical to understanding device operation and to enable scaling of devices below 30 nm in diameter. Here we show that the magnetic reversal mechanism is incoherent and that the switching is thermally nucleated at device operating temperatures. Moreover, we find an intrinsic thermal switching field distribution arising on the sub-nanosecond time-scale even in the absence of size and anisotropy distributions or material defects. These features represent the characteristic signature of the dynamic properties in MTJs and give an intrinsic limit to reversal reliability in small magnetic nanodevices.

## Introduction

Recent advances in low power computing technology have enabled the development of high performance portable computing devices such as smart phones and tablet computers. A limiting factor today for mobile and high performance systems is the power consumed by the main system memory which is based on volatile Dynamic Random Access Memory (DRAM). The volatility arises due to electron leakage, requiring frequent refreshing of the stored data resulting in the memory consuming between 30% and 50% of the total system power^1^. Magnetic RAM (MRAM) is a non-volatile solid state memory technology^2^ based on a magnetic tunnel junction (MTJ) where the data are stored as a magnetic state rather than electrical charge^3–5^. The non-volatile nature of the data removes the need for refreshing the data leading to a large reduction in power consumption as well as higher performance.

CoFeB/MgO/CoFeB MTJs have attracted particular interest due to their high thermal stability, low damping and high tunnel magneto resistance (TMR). Thermal stability is determined by the magnetic anisotropy of the device, which in CoFeB MTJs arises due to hybridisation of the atomic orbitals of the magnetic layer and the MgO interface^4,6,7^. In CoFeB/MgO the anisotropy is sufficient to provide thermal stability and to support an out-of-plane magnetisation. High TMR is achieved because MgO acts as a good spin filtering barrier and the good crystallisation of both CoFeB and MgO preserves the spin polarisation of the electrons crossing the MTJ^4,8–10^. Damping is low due to the weak spin-orbit coupling characterising CoFe-alloys and the good crystalline quality of the film, which is required to reduce the critical current for spin transfer torque (STT) switching^4^.

Despite the promising intrinsic properties of CoFeB/MgO, patterned nanoscale devices introduce many complexities including finite size and surface effects, strong magnetostatic interactions and complex magnetisation dynamics. Previous experimental^11–13^ and micromagnetic studies^14–16^ have concluded that the reversal mechanism is likely to be incoherent due to the large lateral size of the devices. However, the nature of the reversal mechanism and in particular the effects of the localised anisotropy induced at the CoFeB/MgO interface and of the temperature are currently unknown. In addition the role of magnetostatic coupling between the free and pinned layers of an MTJ device is an open question due to the strength of the interactions caused by their proximity. A conventional micromagnetic model approaches the limit of validity at sizes relevant for technological applications of MTJs. The discretisation of the system into micromagnetic cells and the fact that the minimum cell size is around 1 nm^3^ precludes the possibility of taking into account the atomic variation of properties which occurs in these systems whose thickness is of the order of few nanometres, e.g. the fact that the anisotropy is localised at the atomic interface between CoFeB and MgO. In addition, finite temperature effects are poorly described because atomic spin fluctuations are neglected and finite size effects that play an important role in determining the thermal stability of the system for in-plane dimensions below 50 nm, cannot be properly captured. The presence of interfaces causes the reduction of surface coordination and hence loss of exchange bonds at the surface, which leads to lower exchange coupling than in a bulk system. The micromagnetic approach tends to underestimate this effect and often only the dynamics of the free layer is considered. Determining the reversal mechanism is critical in evaluating the thermal stability and the switching time in spin transfer torque MRAM devices. Here we investigate the dynamics of CoFeB/MgO nanodots and MTJs at the atomistic level not constrained by the limits of micromagnetic models. The simulations demonstrate that the magnetisation reversal is incoherent for in-plane dimensions larger than 30 nm and that the switching of the magnetisation is driven by a thermal fluctuations. The atomistic thermal fluctuations at the edge of the device nucleate a domain wall which then propagates through the disk, leading to coercive fields significantly lower than for the case of coherent reversal. This new thermally induced reversal mechanism is a feature of atomistic simulations and are not seen shown by other micromagnetic computational methods. The fact that the switching is thermally driven poses an intrinsic limitation to the deterministic reversal process and reduces the thermal stability for small devices.

## Results

### Atomistic modelling of field induced magnetisation reversal

**Figure 1:**
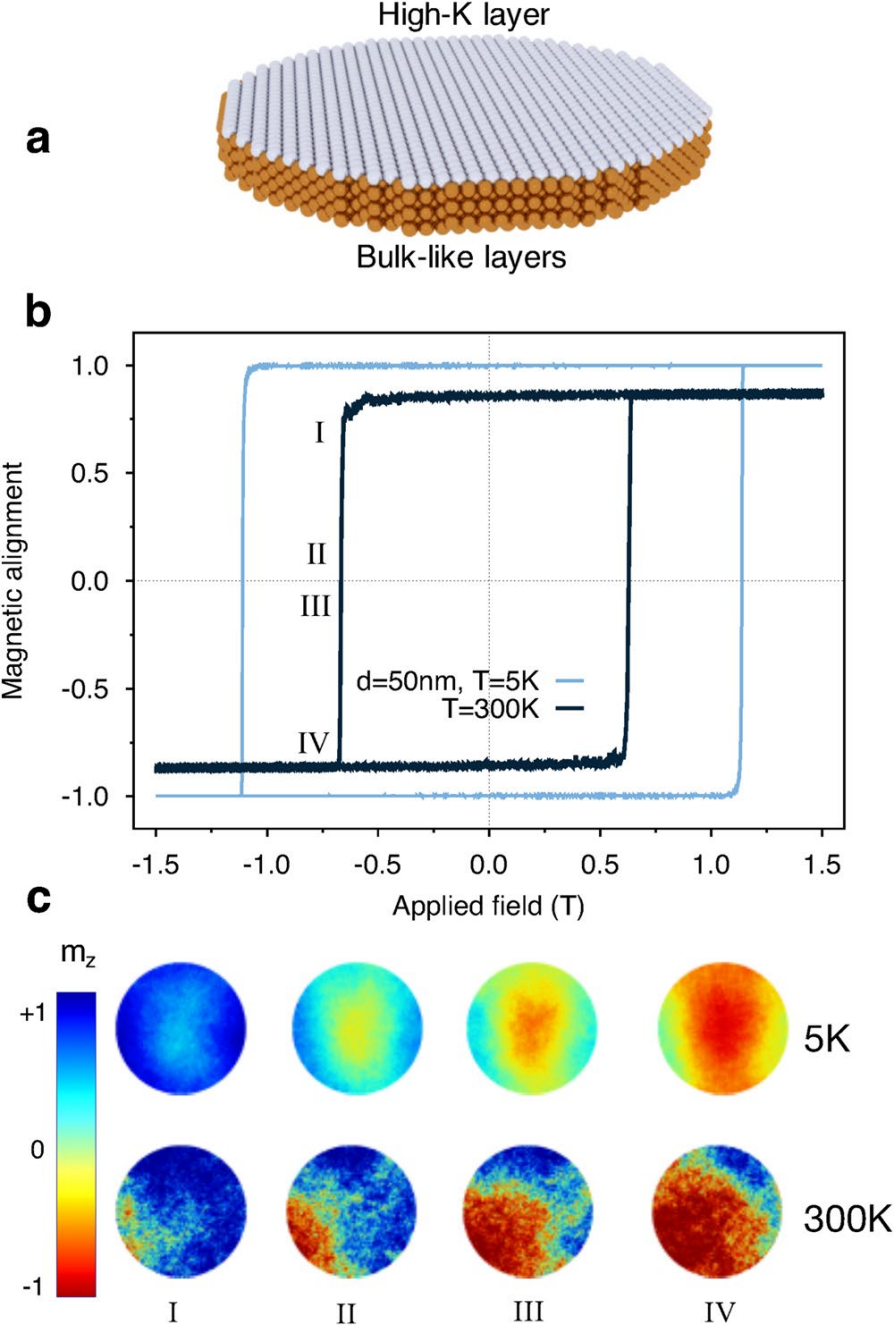
(**a**) Schematic of the simulated system with light spheres representing the high anisotropy layer, and dark spheres representing the bulk-like CoFeB layer. (**b**) Typical simulated easy-axis hysteresis loop for 1 nm thick, 50 nm diameter nanodot at temperatures of 5 K and 300 K. The data shows a large reduction in the coercivity for elevated temperatures due to increased thermal fluctuations, indicating a change in the magnetic reversal mechanism. (**c**) Snapshots of magnetisation reversal at 5 and 300 K for disk of diameter 50 nm and thickness 1 nm. I and IV refer to the top and bottom shoulder of M/*M*_*s*_ vs H curve, respectively. II and III are configurations just before and after the switching, respectively. The colour scheme represents the magnetisation along the easy axis direction (z).

Using an atomistic spin model based on the Heisenberg Hamiltonian as implemented in the vampire software package^17,18^ we simulate the dynamic properties of CoFeB/MgO nanodots, a schematic of which is presented in Fig. 1(a). Thermal effects are included in the model via a Gaussian white noise term whose amplitude is temperature dependent and the magnetostatic contribution using a modified macrocell approach. Details of the model are given in the methods section. We focus on the hysteretic properties of CoFeB/MgO nanodots, in particular investigating the temperature, size and thickness dependence of the reversal mechanism and dynamic coercivity. Finally, we consider the simulations of spin transfer torque switching in an MTJ device including the effects of atomistic thermal fluctuations.

### The role of thermal fluctuations on the coercivity of CoFeB nanodots

We first consider the effects of temperature on the typical hysteresis properties of a nanodot with a diameter of 50 nm, as shown in Fig. 1(b). The first observation is that increased temperatures lead to a large reduction in the coercivity from 1.1 T at 5 K to 0.6 T at 300 K. The temperature variation of intrinsic properties such as the saturation magnetisation and magnetic anisotropy arises naturally from the atomistic simulations, using Monte Carlo methods as outlined in the methods section. This leads to an expected 20% reduction in *H*_*K*_ between zero and 300 K but here we a observe a 45% reduction in the coercivity. This is partially due to the thermally activated transitions over the energy barrier, but also may reflect a change in the magnetic reversal mechanism due to the stronger thermal fluctuations. To investigate the reversal mechanisms we have generated snapshots of the atomic spin configuration during hysteresis for different temperatures, as shown in Fig. 1(c). At a temperature of 5 K the reversal is semi-coherent and nucleated at the centre of the nanodot due to the larger magnetostatic field. This is not generally observed by micromagnetic simulations and demonstrates the importance of the details of the magnetostatic coupling at these device dimensions. At 300 K the reversal is initiated by the nucleation of a small reversed domain at the edge of the nanodot caused by thermally driven spin fluctuations at the edge. At the edge a loss of exchange bonds leads to larger edge spin fluctuations compared with the spins in the middle of the dot. These larger spin fluctuations provide a natural nucleation region at the edges of the nanodot and therefore allow a different reversal mechanism compared to the centre nucleated reversal at low temperatures. Interestingly the small size of the system means that the thermal fluctuations are more important than the variation in the magnetostatic field across the dot diameter, highlighting the importance of including thermal fluctuations and surface effects in the model compared with non-stochastic continuum micromagnetic simulations. Due to the two different nucleation processes at low and high temperatures, the time that is required to reverse the magnetisation varies in the two temperature limits and the switching results faster at 5 K due to the semi-coherent nature of the mechanism.

We note that the thermally nucleated switching we describe here is different from the Sharrock approach^19^ which considers a fixed (coherent) reversal mechanism but with a time dependence of the magnetisation due thermally induced transitions over the energy barrier. In the case of CoFeB/MgO dots the thermal fluctuations lead to a large reduction in the coercivity due to the ability to access a different thermally driven reversal mode. Of course, slower hysteresis loops will likely lead to a further reduction in the coercivity in a similar manner to that of Sharrock due to the increased number of nucleation attempts, but such simulations are currently beyond the time-scales accessible with atomistic models.

Another interesting feature of the hysteresis loop at 300 K in Fig. 1(b) is a slight asymmetry in the coercivity of the ascending and descending branches of the loop. This is due to the thermally nucleated nature of the reversal, leading to an uncertainty in the exact coercivity due to the randomness of the nucleation attempts. There is therefore an intrinsic *thermal* switching field distribution which is independent of defects and variations in the intrinsic properties, but arises solely due to random thermal fluctuations. For larger systems and long time-scales the thermal switching field distribution is not apparent, but for nanoscale MTJs switching in the nanosecond time domain it is a real and important effect and represents the thermodynamic limit of the switching field distribution which cannot be overcome.

### Effect of nanodot diameter on the coercivity and thermal switching field distribution

**Figure 2:**
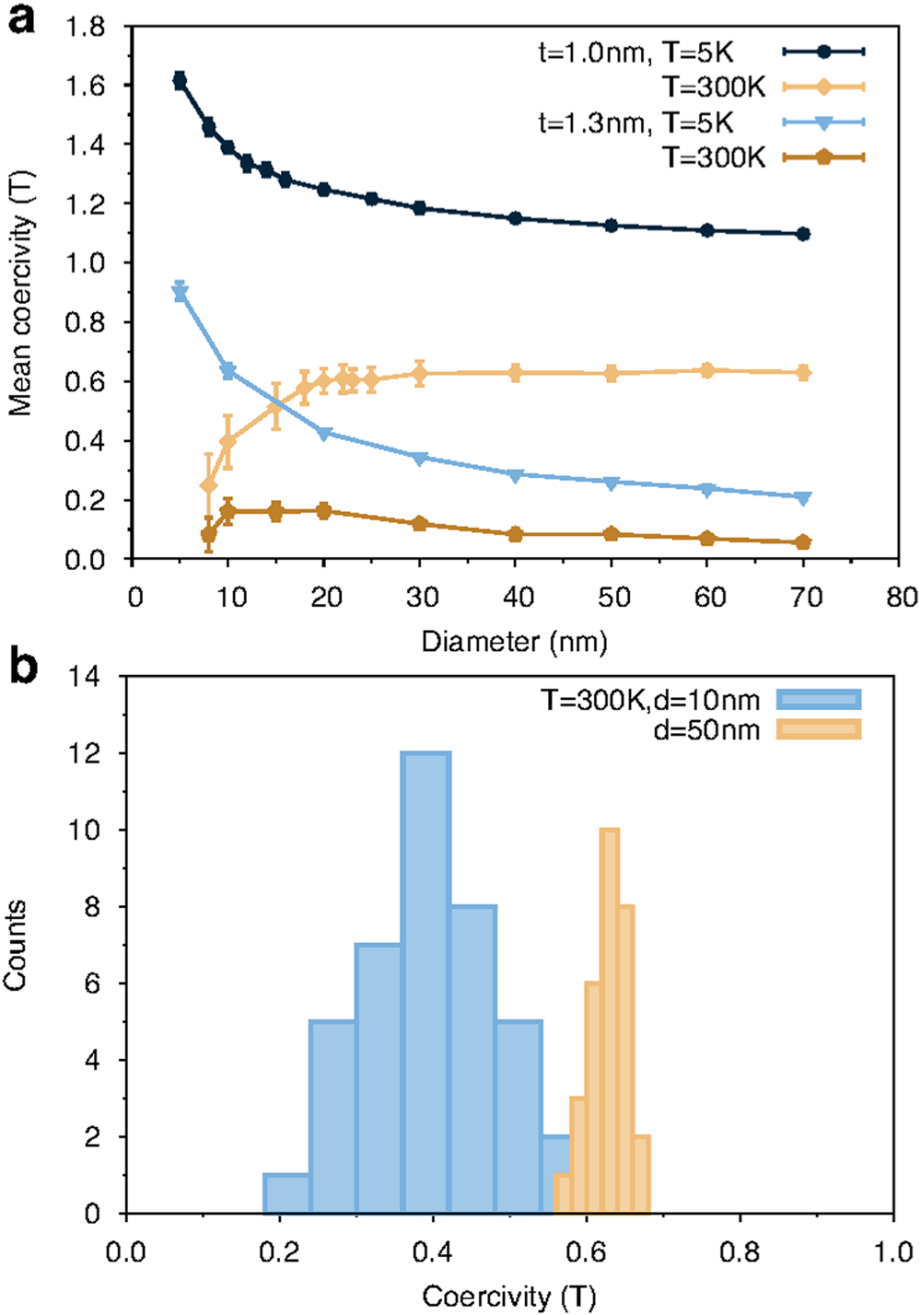
(**a**) Mean coercivity of CoFeB/MgO nanodots as function of disk diameter for thicknesses of 1.0 and 1.3 nm at 5 and 300 K. Error bars show the standard deviation of the statistical distribution. The data shows a constant size dependence for diameters larger than 30 nm because of domain nucleation as reversal mechanism. For smaller diameters, the system becomes thermally unstable and the coercivity reduces at room temperature, while low temperature results in larger stability. (**b**) Calculated switching field distributions at 300 K for 10 and 50 nm nanodots. The data show that reduced nanodot diameters lead to a larger thermal switching field distribution affecting the stability of the magnetisation.

To investigate the effects of nanodot size and temperature on the coercivity and thermal switching field distribution we have performed a systematic study of the hysteretic properties for 1 nm and 1.3 nm thick nanodots, shown in Fig. 2(a). The size dependence of the coercivity is obtained by averaging over a minimum of 30 independent loops for each size, temperature and thickness. The mean coercivity shows a complex temperature and size dependence which is due to different reversal mechanisms and finite effects. Considering first the 1 nm thick nanodots, the coercivity reaches an asymptotic limit for nanodot diameters >20 nm indicative of a nucleation reversal mode at 300 K with a slower approach at 5 K. However the snapshots of the atomic spin configurations support the earlier conclusion of different reversal modes at low and room temperature respectively. At 5 K the nucleation is driven by the variation of the magnetostatic field across the nanodot, which increases with increasing nanodot diameter leading to a slow convergence to a constant nucleation field only seen for larger nanodot diameters (around 100 nm, Supplementary Fig. 3, Supplementary Note 2). Conversely at 300 K the thermal nucleation volume is much smaller and independent of the dot size, and so the coercivity reaches an asymptotic limit at around 20 nm diameter. For dots smaller than 20 nm diameter the temperature has a dramatic effect on the coercivity, showing a large increase at 5 K and large decrease at 300 K respectively. We note that the increase of coercivity with decreasing diameter is indicative that the system has not reached the critical diameter for superparamagnetic behaviour.

For low temperatures the increase in the coercivity with decreasing diameter indicates a transition to coherent reversal (see Supplementary Fig. 2, Supplementary Note 1), where the magnetostatic field no longer dominates the reversal process and the nanodot size approaches the single domain limit ${\delta }_{w}=\pi \sqrt{{A}_{{\rm{s}}}/{K}_{{\rm{eff}}}} \sim 10$ nm. At room temperature the reduction in the coercivity is due to superparamagnetic fluctuations of the magnetisation which due to the small volume lead to switching at fields lower than the intrinsic coercivity. We note here that unlike the work of Brown^20^ there is no peak in the coercivity due to the approximately two-dimensional nature of the nanodots and large anisotropy, hence the direct transition between superparamagnetic and nucleated reversal behaviour as a function of the nanodot size. For dots of diameter smaller than 10 nm the system enters in a single domain limit (in agreement with the estimation of the single domain size *δ*_*w*_) and at room temperature the system becomes unstable due to a transition towards superparamagnetic regime. A similar size dependence is observed in other works^12,21^, although in the latter the investigated system has a lower effective anisotropy. This causes a larger critical single domain size and a less stable system, and therefore larger diameters are used. The 1.3 nm thick nanodots show a similar qualitative behaviour as the 1 nm thick nanodots as a function of the nanodot size, though with a significantly reduced coercivity. The large reduction in the coercivity arises mainly from the reduced anisotropy energy because of its proportionality with 1/thickness. In addition, a change in the magnetostatic energy caused by the increased thickness contributes to the decrease in the coercivity. The combination of these effects reduces the stability of the perpendicular orientation of the magnetisation and therefore increases the stability of nucleated domains under an applied field.

The statistical distribution of the coercivity for different nanodot sizes and temperatures is also strongly size dependent. The extracted switching field distributions (SFD) at room temperature for diameters of 10 and 50 nm and thickness 1 nm are presented in Fig. 2(b). The distributions show a range of switching fields which is much larger for the smaller nanodot size. In the case of our simulations, each nanodot of a given size is identical in terms of the number of atoms and magnetic parameters, but with different pseudorandom number sequence representing the random nature of the thermal noise in the simulations. Therefore, the origin of this distribution is purely the random thermal fluctuations during the reversal process, and hence the distribution is the *thermal* switching field distribution (TSFD)^22^. At the switching field the time scale of the reversal is determined by these random thermal fluctuations, leading to a natural TSFD for a switching process on the time-scale of a few nanoseconds. The TSFD is an intrinsic property of small magnetic elements and cannot be overcome due to its intrinsic thermodynamic origin. We note that the TSFD is also thickness dependent, being narrower for thicker films due to the reduced thermal fluctuations associated with the larger magnetisation volume. Importantly the TSFD intrinsically limits the ability to reliably reverse a nanodot at a given field and time-scale, leading to a natural distribution of switching probability for a finite time and strength of an applied field pulse^21^.

### Switching dynamics of a Magnetic Tunnel Junction device

**Figure 3:**
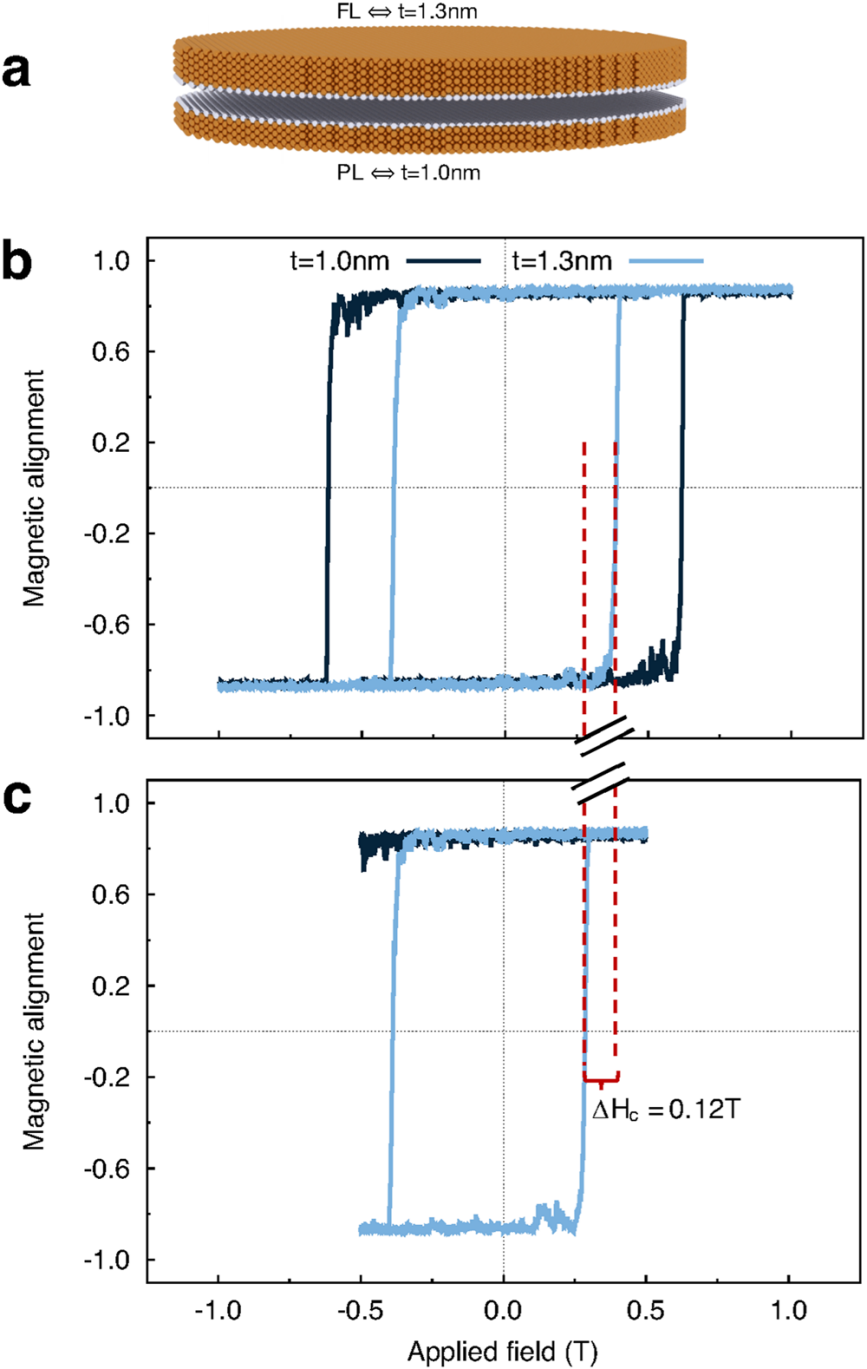
(**a**) Schematic of the simulated MTJ structure: free layer (FL) top and pinned layer (PL) bottom. Major (**b**) and minor (**c**) hysteresis loops for an MTJ of diameter 30 nm at 300 K, the dotted lines mark the coercivity of FL. The major loops show a large enhancement of both layer coercivities due to the coupling to the stray field. The minor loop exhibits a shift of the hysteresis loop due to the asymmetric effect of the pinned layer stray field for descending and ascending branches Δ*H*_*c*_ of 0.12 T, which is larger than the distribution of coercivity of FL.

**Figure 4:**
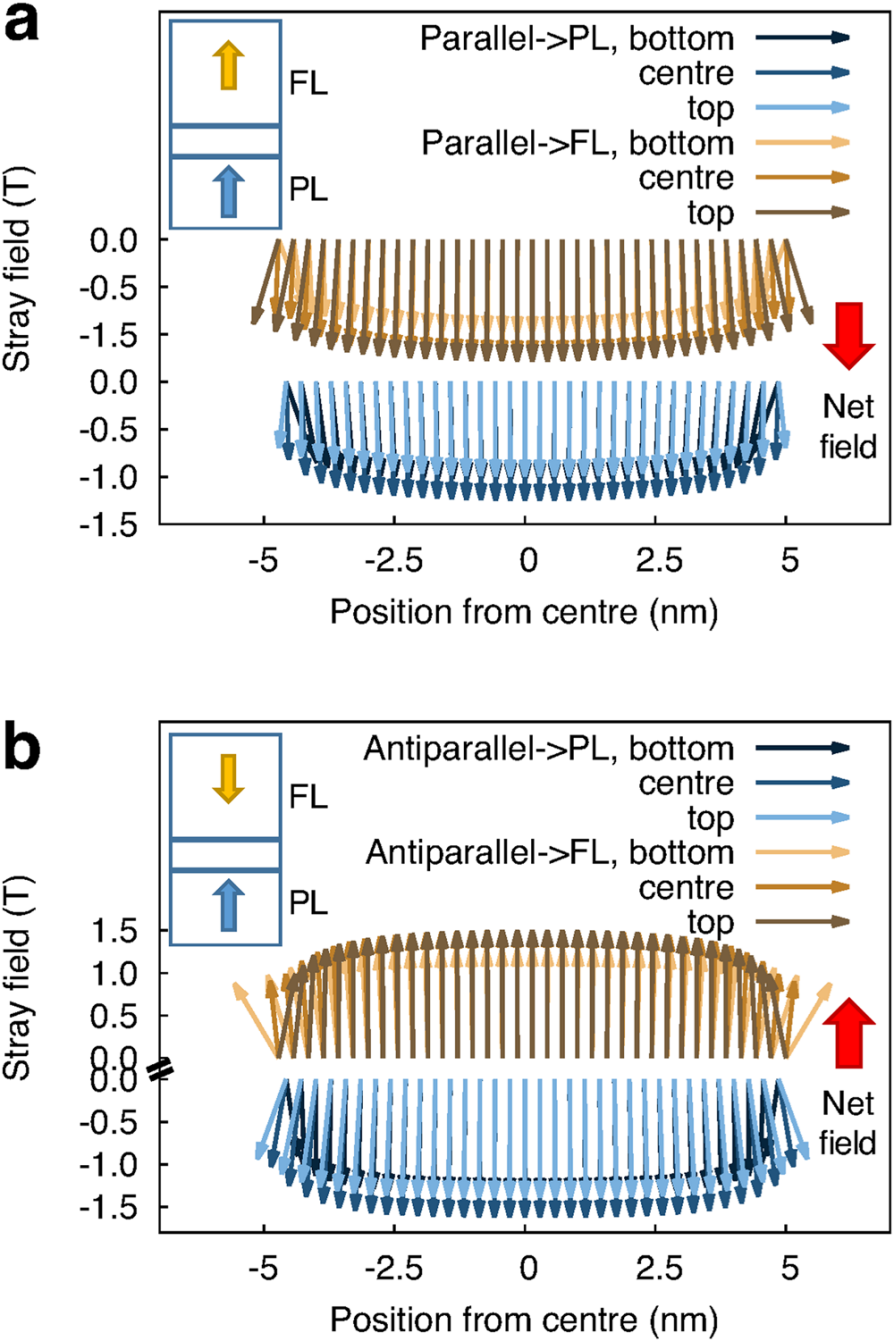
Stray field generated by three atomic layers in PL (black and blue arrows) and FL (yellow and brown arrows) of a 10 nm MTJ in parallel (**a**) and anti-parallel (**b**) configuration as function of position from the centre of the disk. The insets show a schematic of the layer magnetisation and the net average stray field.

So far we have considered the properties of isolated CoFeB/MgO nanodots, however the close proximity of the layers in an MTJ device leads to a significant magnetostatic interaction between the layers. We have investigated the dynamics and magnetisation reversal, including the effects of magnetostatic interactions, in an MTJ structure with dimensions CoFeB(1.0 nm)[PL]/MgO(0.85 nm)/CoFeB(1.3 nm)[FL] and 30 nm diameter, shown schematically in Fig. 3(a). Due to the strong coupling in MTJ, we have modified the usual macrocell approach for the calculation of the magnetostatic field following the approach proposed by Bowden^23^ to obtain exact agreement with the atomic scale dipole-dipole interaction assuming a uniform magnetisation in each cell, a good approximation for our cell size of 1 nm^3^, as discussed in more detail in the methods section. We have calculated major and minor hysteresis loops for the MTJ structure at room temperature as shown in Fig. 3(b) and (c) respectively. We find that the free and pinned layers switch independently and that the reversal mechanism exhibits the same features observed for the individual layers, that of thermally nucleated switching (see Supplementary Figs 4 and 5, Supplementary Note 3). In major loops, compared to the single layer coercivities the magnetostatic coupling in the MTJ tends to stabilize the magnetic structure and enhances the coercivity of both layers compared to the free nanodots of about 0.1 T and 0.2 T for pinned and free layer, respectively. In the minor loop, shown in Fig. 3(c), the free layer exhibits a bias due to the stabilising (destabilising) effect of the magnetostatic field from the pinned layer for the descending (ascending) branches. To quantify the the magnetostatic field from the pinned layer acting on the free layer we have calculated the stray field with atomic resolution as function of position and the net average stray field in Fig. 4, showing the existence of a stabilising (destabilising) field depending on magnetic configuration.

**Figure 5:**
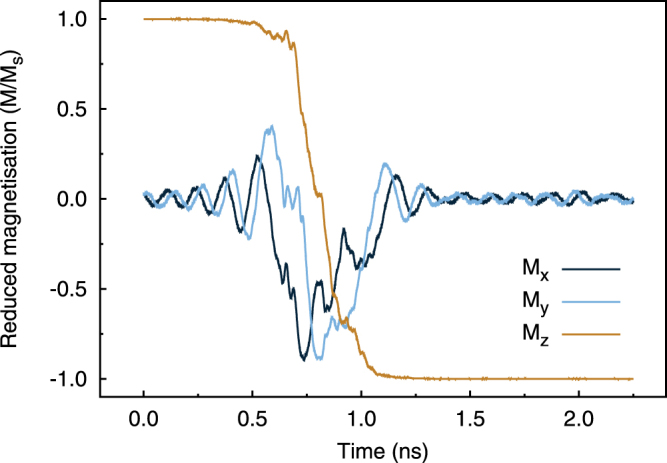
Time evolution of magnetisation at room temperature of a 40 nm diameter MTJ during a simulation of spin transfer torque switching with an injected current density of 1 × 10^11^ Am^−2^. The line colours refer to the different components of the magnetisation.

In the case of MTJ devices the strong coupling of the magnetic layers leads to a a complex change in the magnetic properties such as coercivity. We have also investigated the effect of thermal fluctuations on spin transfer torque switching mechanism following Slonczewski’s approach^24^. Figure 5 shows the time evolution of magnetisation for a MTJ of diameter 40 nm at room temperature. We observe that the magnetisation is reversed in the order of a nanosecond, in agreement with switching times measured experimentally by Devolder *et al*.^25^ and Hahn *et al*.^26^. From the analysis of the spin configurations during the spin transfer torque switching (see Supplementary Movie 1, Supplementary Note 4), a thermally activated incoherent reversal occurring via domain nucleation at the edge of the dot emerges. The edge nucleated nature of the reversal agrees with the reversal mechanism induced by an applied field as discussed previously. A stochastic nature of the magnetisation reversal is also found in the works of Devolder *et al*.^27^ and Hahn *et al*.^26^. There they investigate the magnetisation switching due to spin transfer torque by means of time-resolved measurements and find that the spin transfer torque switching is thermally activated for comparable in-plane dimensions of MTJs. On the other hand, it is not possible to access the reversal mechanism of the magnetisation experimentally for such time-scales and therefore conclusions relative to the switching mechanism are possible only via indirect observations. Devolder *et al*.^27^ find that for 100 nm and smaller MTJs the switching is irreversible with a weak dependence of the switching time on the device area and they explain it assuming nucleation of a domain at the edge of the system which then sweeps through the device. Similar analysis and conclusion are presented by Hahn *et al*.^26^. It is worth pointing out that Hahn *et al*. expect a change in the switching mechanism for diameters of about 50 nm, close to the estimate of the critical diameter for their system. Given our set of parameters, we expect this to occur at smaller dimensions, between 10 and 20 nm, as also shown in the case of hysteresis loops. Therefore, experiments performed on spin transfer torque dynamics for similar MTJ stacks of comparable dimension confirm the stochastic nature of the switching excluding a macro-spin nature of the reversal but rather a non-collinear mechanism characterised by domain wall nucleation and propagation. Nonetheless, we stress that the reversal mechanism we observe is characterised by a precessional motion where both the x and y-components oscillate in time and not by a pure domain sweeping through the system, as it occurs in hysteresis simulations and is assumed experimentally. Hence, atomistic simulations provide an insight into the nature of the reversal mechanism that would not be accessible otherwise experimentally due to the fast time-scale at which the spin torque dynamics occurs and by micromagnetic models because limited to 0 K. Interestingly, no large differences in the mechanism of the switching of the magnetisation at low and high temperature are found. This seems to suggest that the spin torque field acts favouring nucleation modes at the edge of the system and therefore edge nucleation should be expected.

## Discussion and Conclusion

We have investigated the magnetisation reversal mechanism in CoFeB/MgO nanodots and MTJs using an atomistic spin model with the inclusion of thermal and magnetostatic effects. The magnetisation reversal in CoFeB/MgO nanodots and MTJs can be described as thermally nucleated and incoherent at temperatures relevant to device operation, leading to a large reduction in the coercivity compared to a coherent reversal mechanism. The thermal nature of the reversal mechanism is also reflected in the spin transfer torque switching mechanism of MTJ devices, hence affecting the reversal speed. In an MTJ geometry we find that the magnetostatic interaction between the layers leads to a stabilising effect on both the pinned and free layers and causes a shift of the minor hysteresis loop. Our results highlight the importance of considering finite size and thermal fluctuations when modelling such small scale magnetic devices which can have a dominant effect on their reversal mechanisms and physical properties. It is important to note the large difference between the coercivities in our simulation of a perfect nanodot and those measured experimentally, where coercivities are typically ~0.1 T. In our model we have used material parameters derived from experimental measurements of continuous thin films, and as such our simulations represent the best case situation concerning large coercivity. We expect that realistic devices are affected by edge damage and defects which lead to a further reduction of the coercivity. Our results also raise further questions on the role of thermal fluctuations on spin transfer torque switching and the energy barrier in zero field responsible for the thermal stability of MTJs. We expect that the time-scale of the spin transfer torque switching is strongly dependent on the lateral size of the MTJ due to different magnitudes of the thermal fluctuations breaking the magnetic symmetry required for switching and will be the subject of future work.

## Methods

### Atomistic spin model

The simulations were performed using an atomistic spin model where the energy of the system is described by a classical Heisenberg Hamiltonian (${ {\mathcal H} }$)

1 $${\mathscr{H}}=-\sum _{i < j}{J}_{ij}{{\bf{S}}}_{i}\cdot {{\bf{S}}}_{j}-\sum _{i}{k}_{{\rm{u}}}{S}_{i,z}^{2}-\sum _{i}{\mu }_{{\rm{s}}}{{\bf{S}}}_{i}\cdot {{\bf{H}}}_{{\rm{a}}{\rm{p}}{\rm{p}}}+{{\mathscr{H}}}_{{\rm{d}}{\rm{e}}{\rm{m}}{\rm{a}}{\rm{g}}}.$$

where S_*i,j*_ are normalized spin vectors on site *i*, *j* respectively, *J*_*ij*_ is the exchange coupling between spin *i* and *j*, *k*_u_ is the single-ion uniaxial magnetocrystalline anisotropy energy (MAE) constant per site, *μ*_s_ the atomic spin moment, **H**_app_ the applied external field and ${{\mathscr{H}}}_{{\rm{d}}{\rm{e}}{\rm{m}}{\rm{a}}{\rm{g}}}$ the magnetostatic contribution. First and second term on RHS of equation 1 describe a system with nearest neighbours isotropic exchange interactions and uniaxial MAE respectively, while the third term represents the Zeeman interaction with an external field^18^. Given the high computational cost required to calculate calculate the magnetostatic energy due to the long range nature of this interaction and because the fluctuation of the exchange energy are generally larger than that of the magnetostatic energy, the demagnetisation field is computed applying a micromagnetic discretisation of the system into macrocells that are considered as dipoles. Each macrocell *i* has a magnetic moment ${{\bf{m}}}_{i}^{{\rm{mc}}}$ determined by the vector sum of the atomic spin moments inside the cell and position calculated from the magnetic centre of mass of the cell and volume ${V}_{p}^{{\rm{mc}}}$ ^18^. The magnetostatic energy ${{\mathscr{H}}}_{{\rm{d}}{\rm{e}}{\rm{m}}{\rm{a}}{\rm{g}}}$ takes the form $-\frac{1}{2}{\sum }_{p}{{\bf{m}}}_{p}^{{\rm{mc}}}\cdot {{\bf{H}}}_{{\rm{demag}}}^{p}$ with ${{\bf{H}}}_{{\rm{demag}}}^{p}$ the magnetostatic field within the macrocell given by:

2 $${{\bf{H}}}_{{\rm{demag}}}^{p}=\frac{{\mu }_{0}}{4\pi }(\sum _{p\ne q}\frac{3({{\bf{m}}}_{q}^{{\rm{mc}}}\cdot \hat{{\bf{r}}})\hat{{\bf{r}}}-{{\bf{m}}}_{q}^{{\rm{mc}}}}{{r}^{3}})-\frac{{\mu }_{0}}{3}\frac{{{\bf{m}}}_{p}^{{\rm{mc}}}}{{V}_{p}^{{\rm{mc}}}}$$

where *r* is the distance between macrocells *p*, *q* and $\hat{{\bf{r}}}$ is a unit vector pointing along the direction $\vec{pq}$. In equation 2 the first term represents the dipolar field acting on a macrocell *p* due to all the other macrocells, the second accounts for the self-demagnetisation field experienced by the moment of the macrocell ${{\bf{m}}}_{p}^{{\rm{mc}}}$ itself. It is important to note that this approach requires the size of the macrocell used to discretise the system to be much smaller than the system size. We have modified the previously mentioned method for calculating the magnetostatic field to simulate the whole MTJ stack due to the strong coupling between FL and PL and because of the the abrupt variation of the magnetic properties along the vertical direction of the MTJ. Following the method described by Bowden^23^, we have developed a hierarchical approach that allows to achieve atomistic resolution in nearby macrocells. In this method the system is still discretised into macrocells and within each macrocell the magnetisation is averaged, but the self demagnetisation term of the dipole-dipole matrix and the term including the interaction with neighbouring cells are calculated with atomistic resolution with an atomistic dipole-dipole sum. For the interaction with cells located at a larger distance, we can assume that spins inside a cell, provided that the cell size is small, behave as single macro-moment and the interaction between those is calculated disregarding atomistic dipole-dipole expansion. From Fig. 4 it can be seen as this hierarchical approach enables an extremely accurate description of the magnetostatic fields at low computational cost. We note that small macrocell sizes are required for the assumption of uniform magnetisation within a cell to be valid and that the atomistic resolution can be limited to few neighbouring cells at finite temperature due to the fluctuations of the other energy contributions.

The dynamics of magnetisation of CoFeB/MgO nandots and MTJs is determined solving the Landau-Lifshitz-Gilbert equation of motion^18^, given by:

3 $$\frac{d{{\bf{S}}}_{i}}{dt}=-\frac{\gamma }{(1+{\alpha }^{2})}[{{\bf{S}}}_{i}\times {{\bf{H}}}_{{\rm{eff}}}^{{\rm{i}}}+\alpha {{\bf{S}}}_{i}\times ({{\bf{S}}}_{i}\times {{\bf{H}}}_{{\rm{eff}}}^{{\rm{i}}})].$$

*γ* is the gyromagnetic ratio of the electron, *α* the Gilbert damping which describes the relaxation of the atomic spins caused by electron-electron and electron-lattice interactions, **S**_*i*_ is the unitary spin vector on site *i* and ${{\bf{H}}}_{{\rm{eff}}}^{{\rm{i}}}$ is the effective field acting on the spin *i*. The simulations for hysteresis loops are performed in a critical damping regime where *α* = 1 in order to allow a faster relaxation of the magnetisation along the direction of ${{\bf{H}}}_{{\rm{eff}}}^{{\rm{i}}}$, while the mechanism is not affected. The aim of such approach is to obtain a result close to quasi-static hysteresis loops and therefore close to experimental measurements. ${{\bf{H}}}_{{\rm{eff}}}^{{\rm{i}}}$ is obtained differentiating the Hamiltonian 1 with respect to **S**_*i*_. The effect of temperature is introduced by adding a white noise term to **H**_eff_ given the uncorrelated nature of thermal fluctuations on the considered time-scale (≥ns) following the approach proposed by Brown^28^. The thermal field ${{\bf{H}}}_{{\rm{th}}}^{{\rm{i}}}$ is expressed as:

4 $${{\bf{H}}}_{{\rm{th}}}^{{\rm{i}}}={\bf{G}}(t)\sqrt{\frac{2\alpha {k}_{{\rm{B}}}T}{\gamma {\mu }_{{\rm{s}}}{\rm{\Delta }}t}}$$

where **G**(*t*) is a Gaussian distribution in three dimensions, *k*_B_ is the Boltzmann constant, *α* the Gilbert damping, *γ* the gyromagnetic ratio, *T* the temperature, Δ*t* the time step used to integrate the equation of motion and *μ*_s_is the atomic spin moment. The stochastic LLG equation of motion is solved by means of a Heun predictor-corrector algorithm, particularly suitable to deal with stochastic phenomena^18^. The spin transfer torque contribution to the field is included in the LLG dynamics based on the work of Slonczewski^24^ and Fert *et al*.^29^ by adding to the effective field the term:

5 $${\rm{STT}}=a({{\bf{S}}}_{i}\times {{\bf{M}}}_{{\rm{p}}})+b{{\bf{M}}}_{{\rm{p}}}$$

where S_*i*_ is the unitarian spin vector on site *i*, **M**_p_ is the unit vector describing the direction of the injected current and *a,b* are the adiabatic and non-adiabatic spin torque parameters which depend on the applied current density and material properties. As *a* and *b* we extract the values from^29^ that correspond to a similar spin-valve structure and current density of 1 × 10^11^ Am^−2^ and a low damping value *α* = 0.003 is used in spin transfer torque switching simulations. The temperature dependence of static magnetic properties such as the saturation magnetisation *M*_s_(*T*) and the magnetic anisotropy energy *K*(*T*) at a given temperature were calculated using respectively conventional Monte Carlo methods and the Constrained Monte Carlo approach^30^.

### Investigated system

We consider an idealized model where all of the magnetic anisotropy is provided by a single monolayer of CoFeB in contact with the non-magnetic MgO and the other layers contribute no anisotropy. The elemental properties of Fe, Co and B are not considered, but treated as an average magnetic material with zero anisotropy. The atomic structure of CoFeB is modelled as a bcc lattice with lattice constant 2.86 Å and the bulk bcc crystal is cut into the shape of a cylinder of thickness 1.0 and 1.3 nm, representing the pinned layer (PL) and the free layer (FL) for the MTJ respectively as shown in Fig. 1(a). The non-magnetic MgO oxide layer is not included in the simulations explicitly. *Ab-initio* studies^31,32^ suggest that MgO induces a strong interfacial perpendicular anisotropy at the interface CoFeB/MgO and enhances the exchange coupling of Fe and Co sites at the same interface, therefore we model these properties using effective anisotropy and exchange parameters obtained from direct comparison with experiments [Sato *et al*., “Temperature dependent properties of CoFeB/MgO thin films: experiments versus simulations” [submitted 2016]]. The atomic spin moment used for our simulations is *μ*_s_ = 1.60 *μ*_B_ corresponding to M_s_ ~ 1.3 MAm^−1^, close to the experimental value^33^. The value of the atomic spin moment in our simulations is significantly lower than expected experimentally for bulk CoFe or from *ab-initio* calculations of CoFe/MgO, where values close to 2.5 *μ*_B_ are found^34,35^. Experimentally the CoFeB/MgO system is known to have a perpendicular orientation for effective thicknesses less than 1.2–1.3 nm^6,11,33,36–40^ and hysteresis simulations for different atomic moments (Supplementary Fig. 1, Supplementary Methods) confirm that an effective atomic moment less than 2 *μ*_B_ is required to have perpendicular orientation of the magnetisation and square loops. The physical origin of the reduced saturation magnetisation is likely due to a combination of the presence of non-magnetic Boron and the possibility of structural defects in the material. The effect of the demagnetising field is included in the calculations using a macrocell approach^17^ with a cell size of 1 nm. The used parameters are reported in Table 1.

**Table 1:** Simulation parameters for the investigated systems.

	CoFeB(@interface)	CoFeB(bulk)	Unit
*J*_*ij*_	1.547 × 10^−20^	7.735 × 10^−21^	J link^−1^
*μ*_s_	1.60	1.60	*μ*_B_
*k*_u_	1.35 × 10^−22^	0.0	J atom^−1^

In the hysteresis loop calculations we use a critical damping and calculate a complete hysteresis cycle over 20 ns with an effective field rate of 0.3 T ns^−1^ to minimize the effects of enhanced coercivity caused by fast field sweep rates.

## Electronic supplementary material

Supplementary movie

Supplementary information
