# Supplementary material for: Thermally nucleated magnetic reversal in CoFeB/MgO nanodots
Source: Sci Rep. 2017 Dec 1;7:16729. doi: 10.1038/s41598-017-16911-3 (PMC5711886; doi:10.1038/s41598-017-16911-3)
Supplement: Supplementary file 2 — Supplementary information [file 41598_2017_16911_MOESM2_ESM.pdf]

Supplemental Materials: Thermally nucleated magnetic reversal in CoFeB/MgO nanodots

Andrea Meo[1], Phanwadee Chureemart[2], Shuxia Wang[3], Roman Chepulskyy[3], Dmytro Apalkov[3], Roy W. Chantrell[1] and Richard F. L. Evans[1]

1 Department of Physics, University of York, York, YO10 5DD, UK
2 Computational and experimental magnetism group, Department of Physics, Mahasarakham University, Mahasarakham, Thailand
3 Samsung Electronics, Semiconductor R&D Center (Grandis), San Jose, CA 95134, USA

Supplementary Figures 1-5

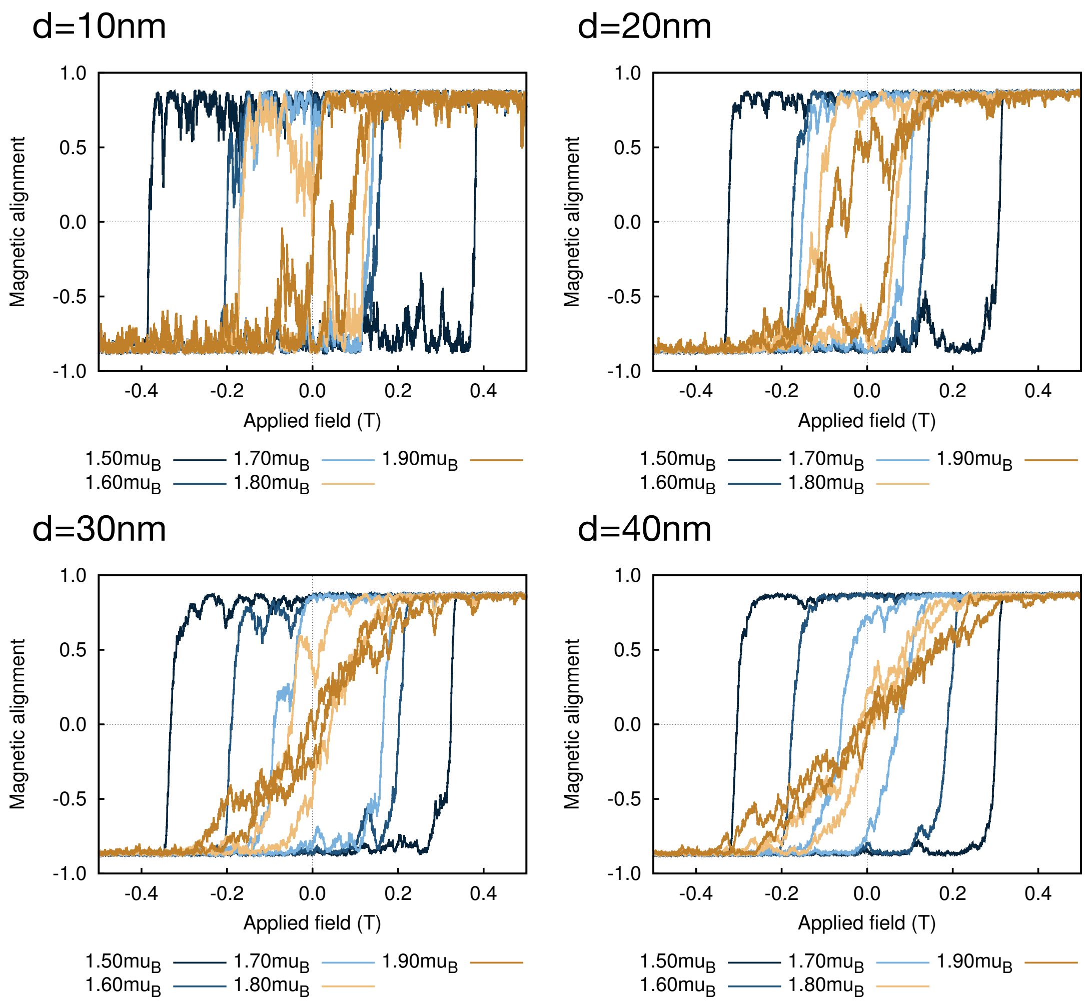

Supplementary Fig. 1: Hysteresis loops performed at room temperature for nanodots of thickness 1.3 nm and diameter 10, 20, 30, 40 nm as function of atomic magnetic moment (μ_s), from 1.5 to 1.9 μ_B. Colours refer to different μ_s values.

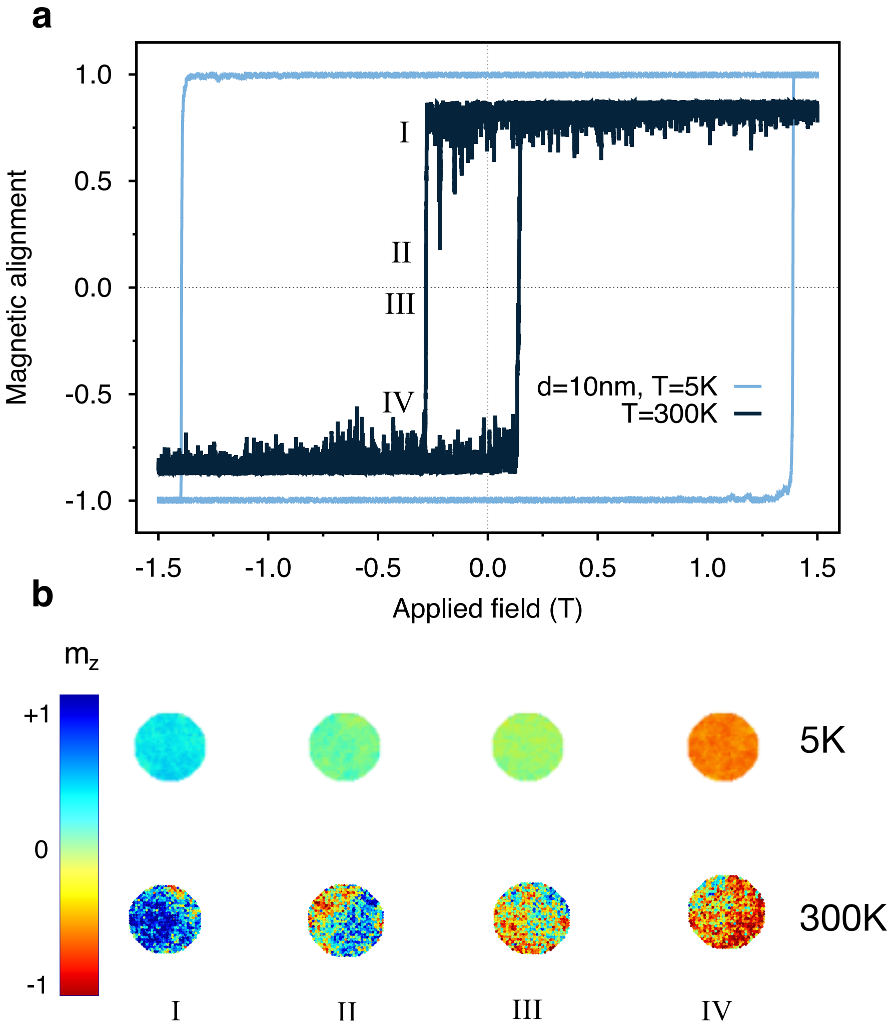

Supplementary Fig. 2: (a) Typical simulated easy-axis hysteresis loop for 1 nm thick, 10 nm diameter nanodot at temperatures of 5 K and 300K. The data show a large reduction in the coercivity for elevated temperatures due to increased thermal fluctuations, indicating a change in the magnetic reversal mechanism. (b) Snapshots of magnetisation reversal at 5 and 300 K for a disk of diameter 10 nm and thickness 1 nm. I and IV refer to the top and bottom shoulder of M/M_s vs H curve, respectively. II and III are configurations just before and after the switching, respectively. The colour scheme represents the magnetisation along the easy axis direction (z).

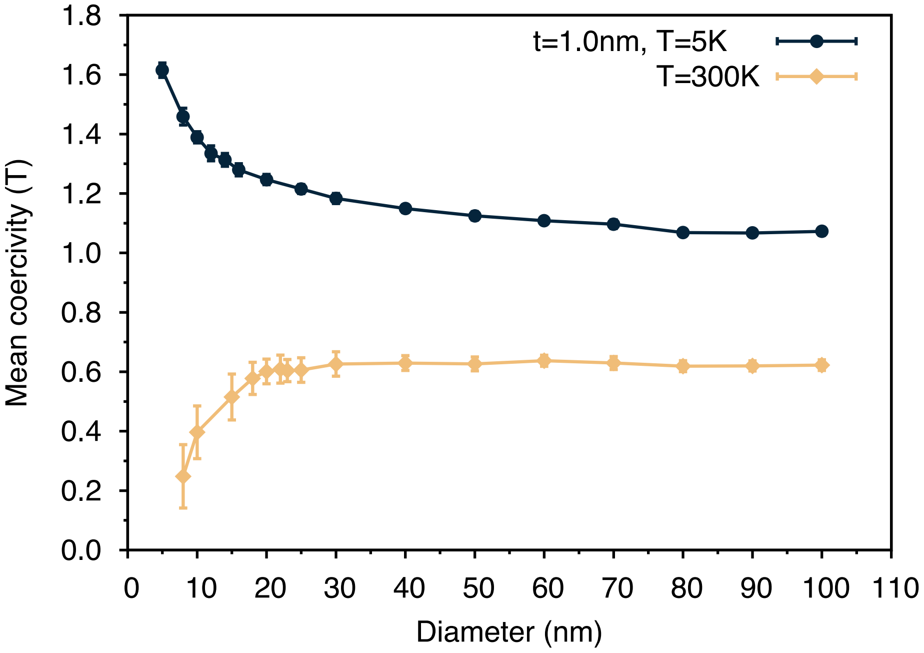

***Supplementary Fig. 3**:* Mean coercivity as function of disk diameter for nanodots of thickness of 1.0 nm at 5 (black dots) and 300 K (yellow diamonds). Error bars show the standard deviation of the statistical distribution.

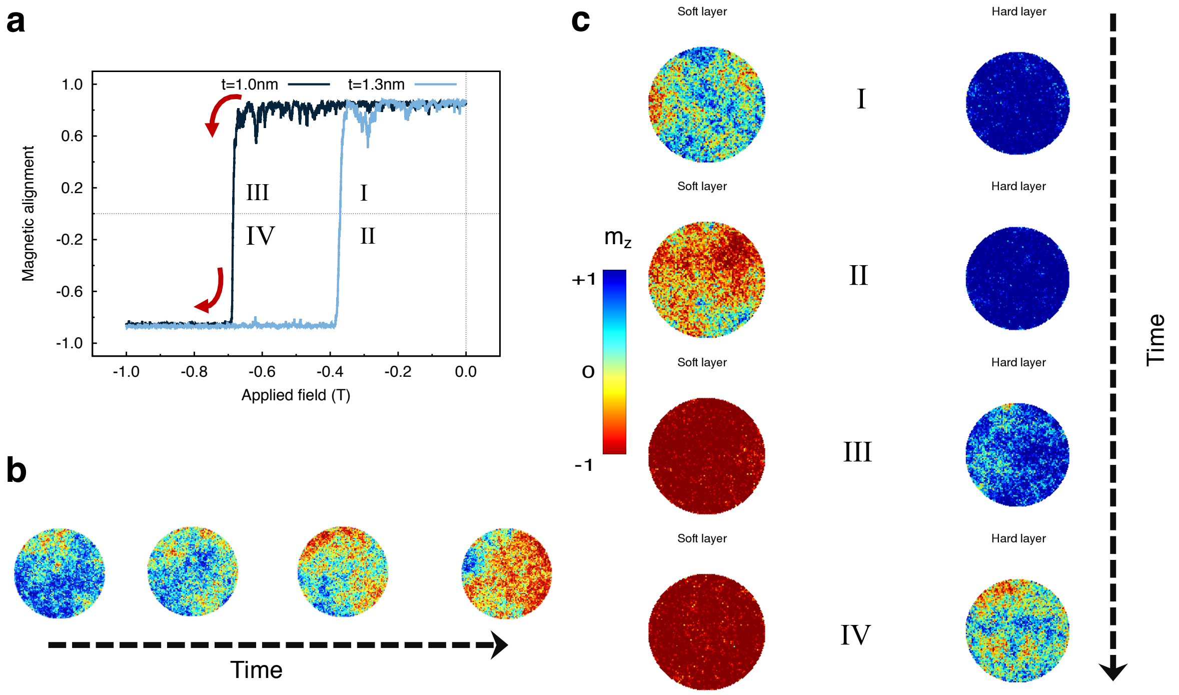

Supplementary Fig. 4: (a) Part of major loop from which the snapshots are taken. The roman numbers represent the field points at which the snapshots are taken. (b) Snapshots of magnetisation reversal at 300 K for a nanodot of diameter 20 nm and thickness 1.0 nm. (c) Snapshots of magnetisation reversal at 300 K for an MTJ of diameter 20 nm during a major loop. Left and right dots represent the free and pinned layer, respectively and roman numbers refer to the field points in (a). The colour scheme represents the magnetisation along the easy axis direction (orthogonal to the dot).

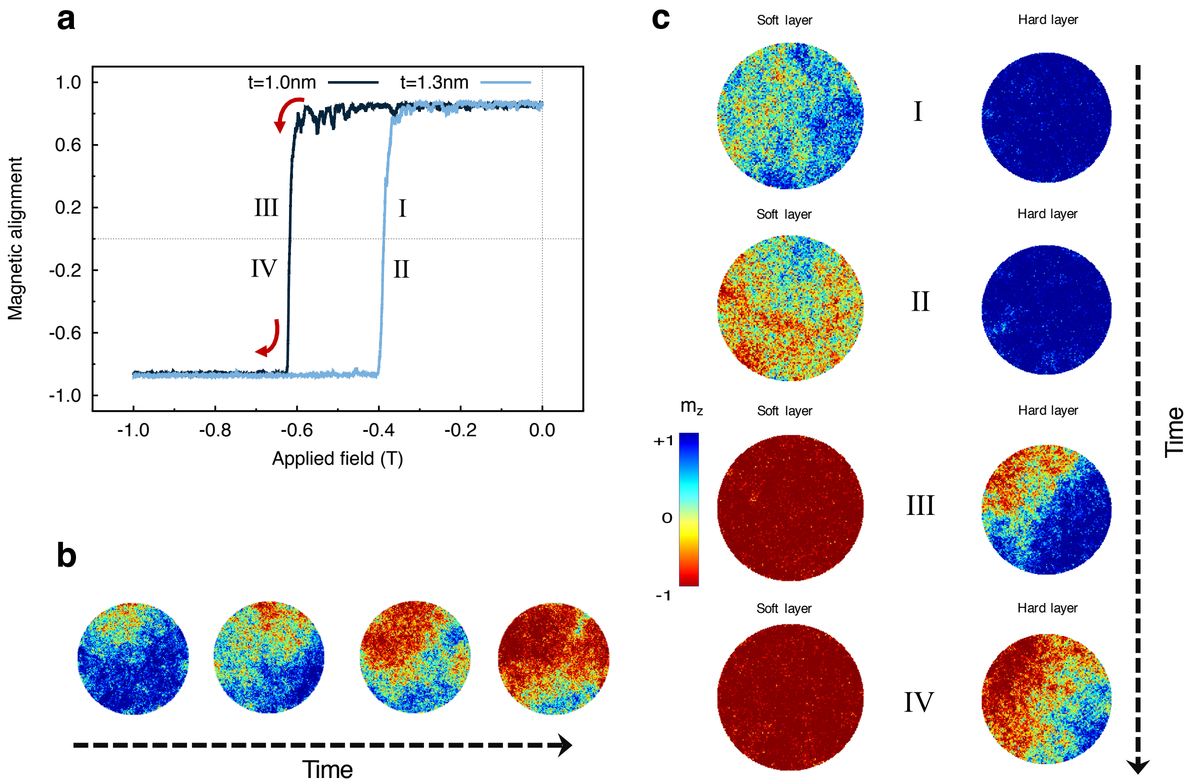

Supplementary Fig. 5: (a) Part of major loop from which the snapshots are taken. The roman numbers represent the field points at which the snapshots are taken. (b) Snapshots of magnetisation reversal at 300 K for a nanodot of diameter 30 nm and thickness 1.0 nm. (c) Snapshots of magnetisation reversal at 300 K for an MTJ of diameter 30 nm during a major loop. Left and right dots represent the free and pinned layer, respectively and roman numbers refer to the field points in (a). The colour scheme represents the magnetisation along the easy axis direction (z).

Supplementary movie 1

Movie showing the evolution of the z-component of the magnetisation during the spin transfer torque switching for a MTJ of diameter 40 nm at room temperature under the application of a current density of 10^{11} Am^{-2}. The colour scheme represents the magnetisation along the easy axis direction (z): blue = spin-up, green = in-plane, red = spin-down.

Supplementary methods

Dependence of coercivity on magnetic moment

Supplementary Fig. 1 shows hysteresis loops performed at room temperature for nanodots of thickness 1.3 nm at room for various diameters as a function of the magnetic moment. Loops for systems characterized by a magnetic moment of 1.9 μ_B and larger show that the magnetisation lies in-plane. For smaller values an out-of-plane character can be observed. In this range, a magnetic moment of 1.6 μ_B gives loops that are square and stable as seen in experiments.

Supplementary Note 1: Effect of temperature on coercivity for small dots

Supplementary Fig. 2 (a) shows the hysteresis loops at 5 K and 300 K for 10 nm dots. A net reduction of the coercivity at room temperature emerges compared with 50 nm dots as well as larger thermal contribution which results in a pronounced asymmetry of the two branches and increase in the noise. From the analysis of the spin configurations in **Supplementary Fig. 2** (b) the reversal appears dominated by fluctuation of the magnetisation due to the small volume. At low temperature the system exhibits high coercivity and the reversal mechanism becomes quasi coherent. As observed for larger dots, the hysteresis loops are symmetric and the thermal fluctuations of the magnetisation become negligible. We point out that even at low temperature the coercivity is expected to decrease as the diameter of the dots is further decreased, due to loss of thermal stability.

Supplementary Note 2: Dependence of average coercive field on diameter for large dots

We calculate the average coercive field for large dots (≥ 70nm) at 300 K and 5 K as function of diameter. The result is shown in **Supplementary Fig. 3**. In the low temperature case the average coercive field tends to an asymptotic value for dots larger than 60-70 nm and the reversal mechanism is non-uniform when the demagnetisation field of the nanodots approaches the saturation limit of a thin film yielding to a constant nucleation field. At room temperature the average coercivity reaches a limit value for nanodots diameters larger than 20 nm, as discussed in relation to Fig. 2.

Supplementary Note 3: Magnetisation reversal in MTJ

We have calculated the magnetic properties of an MTJ. A branch of a major hysteresis loop is shown in **Supplementary Fig. 4** (a), where both the free (soft) and pinned (hard) layers switch their magnetisation. It can be seen that the reversal modes of both layers show the same feature and do not reverse independently. This can be seen by comparing the magnetisation reversal configurations with the switching of the individual layers. **Supplementary Fig. 4** (b) presents the snapshots of the switching of the magnetisation for a nanodot of the same diameter and thickness 1.0 nm. From the comparison, we can observe as the mechanism of the reversal is the same: the magnetisation reversal occurs via thermal activation, therefore confirming that in our simulation the reversal mechanism of each single layer constituting the MTJ is not affected by the stacking. In **Supplementary Fig. 5** (a) the same results obtained for a MTJ of diameter 30 nm are presented. Conversely from the previous case, in **Supplementary Fig. 5** (b) we clearly observe edge nucleation, in agreement with the analysis proposed for the single layers at room temperature. A comparison of the hysteresis branch presented in (a) show how for 30 nm the magnetisation results more thermally stable close to the nucleation field.

Supplementary Note 4: Spin transfer torque switching in MTJs

We simulate the spin transfer torque switching of a MTJ with diameter 40 nm at room temperature under the application of a current density of 10^{11} Am^{2}.

The ferromagnetic layers are described with low damping (α=0.003) and the spin torque switching is modelled such that it is originated at the interface CoFeB(Free layer)/MgO. Supplementary Movie 1 shows a movie describing the spin configurations of the free layer during the switching, where the colours describe the z-component of the magnetisation. A clear domain wall edge nucleation and subsequent propagation emerge from the animation. The stochastic nature of the thermal fluctuations is responsible for the edge nucleation allowing nucleation sites localized at the edge of the system.
